# Structural Performance Analysis of a Novel Pyramidal Cellular Core Obtained through a Mechanical Expansion Process

**DOI:** 10.3390/ma13194264

**Published:** 2020-09-24

**Authors:** Mihaela Iftimiciuc, Simona Lache, Per Wennhage, Marian Nicolae Velea

**Affiliations:** 1 Department of Mechanical Engineering, Transilvania University of Brasov, 500036 Brasov, Romania; mihaela.iftimiciuc@unitbv.ro (M.I.); slache@unitbv.ro (S.L.); 2 Department of Engineering Mechanics, KTH Royal Institute of Technology, SE-100 44 Stockholm, Sweden; wennhage@kth.se

**Keywords:** pyramidal cellular core, out-of-plane compression, mechanical expansion, analytical model

## Abstract

Stiff and strong yet lightweight cellular structures have become widely designed and used as cores for the construction of sandwich panels to reach high stiffness and strength to weight ratios. A low-density pyramidal cellular core has been proposed for investigation in this work. The novel core is manufactured from stainless steel sheet type 304 through a mechanical expansion procedure which is described in detail. The out-of-plane stiffness and strength performance is estimated by an analytical model which is successfully validated through experimental tests. A comparative study with other existing cellular core configurations made from other materials indicates an average performance behavior for the investigated structure. However, potential for a further structural performance increase is observed and discussed

## 1. Introduction

Over the past few decades, the use of periodic cellular cores for sandwich structures has become a widely used approach in the context of structural weight reduction and energy saving. The concept has been proven to provide increased stiffness and strength as well as impact energy absorption capabilities, in industries such as aerospace, automotive and naval [1].

Honeycomb cellular cores are the most common choice when it comes to high-performance applications, but the associated manufacturing process remains complex [2,3]. In addition to this, due to their closed-cell structure, they trap moisture, which may lead to internal corrosion and face sheet de-bonding [4]. The intensive research conducted in this field proposes a wide range of low-density cellular cores developed as an alternative to the honeycomb. These range from simple manufactured structures and low structural performance ones to more sophisticated manufacturing procedures and high-performance ones, depending on the targeted application [3]. On the one hand, the corrugated structures represent one of the cores easiest to manufacture, but this does not provide the best solution with respect to stiffness and strength to weight ratios. However, methods for improving their structural behavior have been proposed by considering the hierarchical structure concept [5,6].

On the other hand, lattice-truss cellular cores have been considered more and more in recent years, due to their increased structural performance [7,8,9]. These also offer the possibility of integrating multifunctional characteristics due to the open-space geometry; however, most of the corresponding manufacturing methods are not attractive for bulk and mass production. Research has been carried out to simplify the manufacturing process of pyramidal lattice structures [10] and triangular corrugated structures [11,12] where solutions have been proposed for structures made of thermoplastic composites.

More recently, additive manufacturing technologies bring total freedom with respect to the cellular configuration, however not yet to the type of material that can be used [13,14,15,16]. In addition, additive manufacturing is not yet cost effective for bulk and mass production.

Within this context, a novel pyramidal cellular core is obtained within this study. The stiffness and strength of the newly developed structure is evaluated both theoretically and experimentally in out-of-plane compression. The results obtained are evaluated through a comparison with other types of cellular configurations.

## 2. Cellular Structure and Its Manufacturing Principle

**Figure 1:**
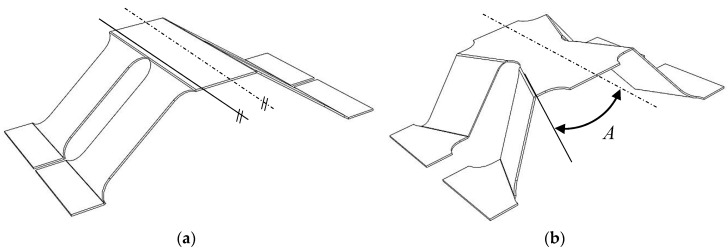
Schematic representation of the cellular structure: (**a**) perforated trapezoidal corrugation; (**b**) mechanically expanded perforated trapezoidal corrugation resulting in a pyramidal structure.

The cellular structure developed in this research is derived from one that is perforated trapezoidal corrugated, with an addition of an internal angle *A*, as shown in Figure 1.

**Figure 2:**
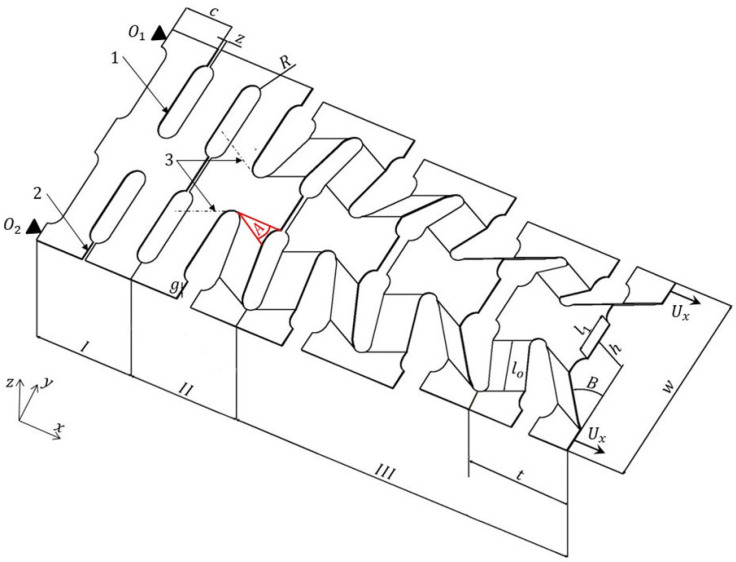
Key steps in the manufacturing process of the pyramidal cellular structure. The parameters defining the structure are: *c*—base of the cell’s arm, *z*—thickness of the cutting tool, *R*—radius of the perforation, *g*—thickness of the base material, *A*—expansion angle of the structure, *t*—width of the expanded structure, *l_o_*—strut length, *l*_1_—distance between the perforations, *h*—height of the expanded unit cell, *B*—inclination of the strut, and *w*—length of the expanded unit cell.

**Figure 3:**
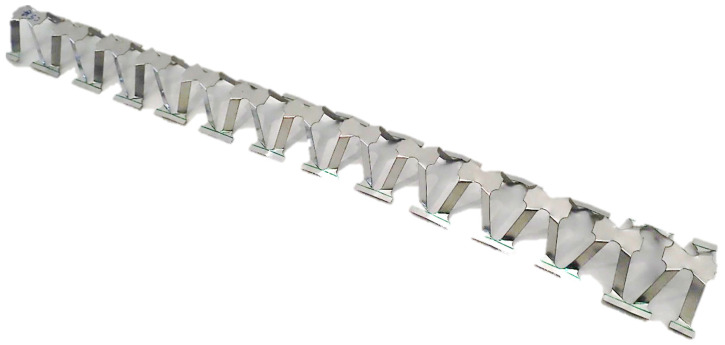
A strip of mechanically expanded perforated corrugated structure—pyramidal cellular structure.

In a continuous manufacturing process, Figure 2, the stages can be described as follows: a series of specific perforations are generated on a flat sheet metal resulting in a profile numbered 1, 2 (zone I). Successively, a pattern of bends, numbering 3, is performed with the purpose of better guidance of the expansion process (zone II). The sheet metal is then fixed at one end (points O_1_ and O_2_) while at the opposite end a specific displacement *U_x_* is applied along the direction of expansion. The expansion process is stopped when the cells reach the desired inclination angle *B*. An example of the manufactured mechanically expanded perforated corrugated structure is shown in Figure 3.

The interdependence of the geometric parameters for the investigated pyramidal structure are indicated in Table 1.

## 3. Analytical Model for Stiffness and Strength in Out-of-Plane Compression

**Figure 4:**
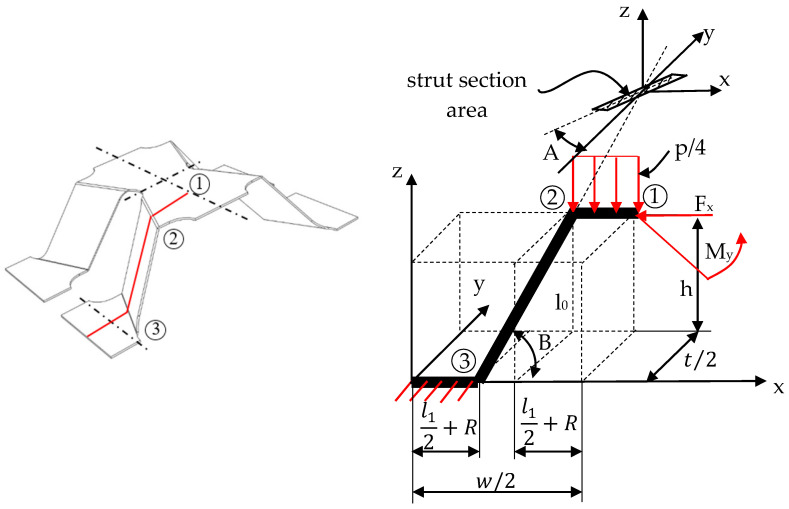
Quarter of unit cell under out-of-plane compression loading.

Due to the symmetric topology of the structure, a quarter of the unit cell has been taken into consideration for developing the analytical model, as shown in Figure 4. It consists of two segments, representing the free members influenced by the external forces acting on the system. The loads and boundary conditions consist of applying a uniformly distributed line pressure *p* on segment 1–2 while setting to zero all DOF’s in point 3. The line pressure imposed on the model in the z direction generates the vertical displacement *δ_z_*.

The resulting displacement *δ_z_* is calculated based on Castigliano’s second theorem [17] which states that, for linear elastic deformations, the displacement *δ_j_* of the point of application *j* of the force *F_j_* along the direction of *F_j_* can be determined as:

(1) $$\delta_{j}=\frac{\partial U}{\partial F_{j}}$$

where *U* is the strain potential energy stored in a volume *V* of a body in equilibrium conditions, and can expressed as [18]:

(2) $$U=\int_{V}\frac{\sigma \varepsilon }{2}dV+\int_{V}\frac{\tau \gamma }{2}dV$$

where σ is the normal stress, τ is the shear stress, ε is the normal strain and γ represents the shear strain.

When the inner forces consist of bending moments and tangential forces, by considering that the material complies with Hooke’s law (σ=E·ε), Equation (2) becomes:

(3) $$U=\int_{V}\frac{\sigma^{2}}{2E}dV+\int_{V}\frac{\tau^{2}}{2G}dV$$

For a Euler beam of length *l*, Equation (3) may be rewritten as:

(4) $$U=\int_{l}\frac{M^{2}}{2EI}dx+\int_{l}\frac{T^{2}}{2GA}dx$$

By replacing U from Equation (4) to Equation (1), we obtain:

(5) $$\delta_{j}=\frac{\partial }{\partial F_{j}}\int_{l}\frac{M^{2}}{2EI}dx+\frac{\partial }{\partial F_{j}}\int_{l}\frac{T^{2}}{2GA}dx$$

This equivalent model formulation was successfully applied to several types of corrugations [19,20] and to expanded hexagonal structures [21].

For the pyramidal structure (Figure 4), the calculated displacement *δ_z_* is:

(6) $$\delta_{z}=p\left(\frac{2R+l_{1}}{32Gcg}-\frac{12l_{0}{}^{2}\left(\frac{\cos\left(B\right)\left(R+\frac{l_{1}}{2}\right)\left(\frac{R}{2}+\frac{l_{1}}{4}\right)}{32}+\frac{\cos\left(B\right)\left(R+\frac{l_{1}}{2}\right)\left(\frac{R}{4}+\frac{l_{1}}{8}\right)}{8}\right)+\frac{l_{0}{}^{3}\cos{\left(B\right)}^{2}\left(R+\frac{l_{1}}{2}\right)}{4}++3l_{0}\left(R+\frac{l_{1}}{2}\right)\left(\frac{R}{2}+\frac{l_{1}}{4}\right)\left(\frac{R}{4}+\frac{l_{1}}{8}\right)}{Eg^{3}\left(2R-c\right)}\right)+\;\frac{12l_{0}{}^{2}\left(\frac{M_{y}\cos\left(B\right)}{8}+\frac{F_{x}\cos\left(A\right)\sin\left(B\right)\left(\frac{R}{4}+\frac{l_{1}}{8}\right)}{2}+12l_{0}M_{y}\left(\frac{R}{4}+\frac{l_{1}}{8}\right)+F_{x}l_{0}{}^{3}\cos\left(A\right)\cos\left(B\right)\sin\left(B\right)\right)}{Eg^{3}\left(2R-c\right)}-\frac{3M_{y}{\left(R+\frac{l_{1}}{2}\right)}^{2}}{2Ecg^{3}}-\frac{F_{x}l_{0}\cos\left(A\right)\cos\left(B\right)\sin\left(B\right)}{4Gg\left(2R-c\right)}$$

where:

$M_{y}=\frac{p2E\left(6R^{2}g^{2}\left(cl_{1}+2cl_{0}-l_{1}{}^{2}\right)+4R^{3}g^{2}\left(c-3l_{1}\right)+Rg^{2}\left(12\cos\left(B\right)l_{0}{}^{2}c-12cl_{0}l_{1}-l_{1}{}^{3}+6cl_{1}{}^{2}\right)-8R^{4}g^{2}\right)}{96\left(ERg^{2}\left(2c-4Rl_{1}+2cl_{0}+cl_{1}\right)+Gl_{0}\left(2cl_{0}{}^{2}16R^{2}l_{0}+8Rcl_{0}-8Rl_{0}l_{1}+4cl_{0}l_{1}\right)\right)}+\;\frac{pG\left(8Rl_{0}l_{1}\left(3cl_{0}{}^{2}-l_{0}l_{1}{}^{2}+3cl_{0}l_{1}\right)-64R^{2}l_{0}{}^{2}+24R^{2}l_{0}\left(cl_{0}{}^{2}+2l_{0}l_{1}{}^{2}2cl_{0}l_{1}\right)+cl_{0}l_{1}\left(4l_{0}l_{1}+6l_{0}{}^{2}l_{1}\right)\right)}{96\left(ERg^{2}\left(2c-4Rl_{1}+2cl_{0}+cl_{1}\right)+2Gl_{0}{}^{2}\left(cl_{0}16R^{2}+4Rc-4Rl_{1}+2cl_{1}\right)\right)}$
$F_{x}=\frac{p2Eg^{2}\cos\left(B\right)\left(2R\left(2R-c+l_{1}\right)-\left(2cl_{0}-cl_{1}\right)\right)}{8\cos\left(A\right)\sin\left(B\right)\left(\left(Eg^{2}\left(2Rc-4R^{2}-2Rl_{1}+2cl_{0}+cl_{1}\right)\right)+2G\left(cl_{0}{}^{3}-8R^{2}l_{0}{}^{2}+4Rcl_{0}{}^{2}-4Rl_{0}{}^{2}l_{1}+2cl_{0}{}^{2}l_{1}\right)\right)}+\;\frac{pG\left(-16R^{4}l_{0}-8R^{3}l_{0}\left(3l_{1}+c+4l_{0}\cos\left(B\right)\right)-4R^{2}l_{0}\left(8\cos\left(B\right)l_{0}l_{1}-4\cos\left(B\right)l_{0}c+3l_{1}{}^{2}-3cl_{1}\right)\right)}{8\cos\left(A\right)\sin\left(B\right)\left(\left(Eg^{2}\left(2Rc-4R^{2}-2Rl_{1}+2cl_{0}+cl_{1}\right)\right)+2G\left(cl_{0}{}^{3}-8R^{2}l_{0}{}^{2}+4Rcl_{0}{}^{2}-4Rl_{0}{}^{2}l_{1}+2cl_{0}{}^{2}l_{1}\right)\right)}+\;\frac{pG\left(-2Rl_{0}\left(2\cos\left(B\right)cl_{0}{}^{2}-4\cos\left(B\right)l_{0}l_{1}{}^{2}+8\cos\left(B\right)cl_{0}l_{1}-3l_{1}{}^{2}+3cl_{1}{}^{2}\right)\right)}{8\cos\left(A\right)\sin\left(B\right)\left(\left(Eg^{2}\left(2Rc-4R^{2}-2Rl_{1}+2cl_{0}+cl_{1}\right)\right)+2G\left(cl_{0}{}^{3}-8R^{2}l_{0}{}^{2}+4Rcl_{0}{}^{2}-4Rl_{0}{}^{2}l_{1}+2cl_{0}{}^{2}l_{1}\right)\right)}$

The effective strain is therefore obtained as:

(7) $$\varepsilon_{z}=\frac{\delta_{z}}{h}$$

where *h* is representing the height of the structure (Table 1).

The effective stress acting on the structure is calculated as:

(8) $$\sigma_{z}=\frac{pl_{1}}{A_{s}}$$

where $A_{s}=wt/4$ represents the compressive area of the structure, with *w* and *t* detailed in Table 1.

The effective stiffness parameter is further on calculated as:

(9) $$E_{z}=\frac{\sigma_{z}}{\varepsilon_{z}}$$

By substituting Equations (7) and (8) into Equation (9), it yields the expression for the out-of-plane compressive stiffness.

The out-of-plane compressive strength model of the investigated structure is developed by assuming the Euler buckling failure mode of the struts. The critical buckling load of the strut can be written as:

(10) $$F_{cr}=\frac{\pi^{2}EI_{2–3}}{4{\left(0.6l_{0}\right)}^{2}}$$

where $I_{2–3}$ represents the cross-section moment of inertia of the strut.

An effective length factor of 0.6 was used as being the average value between fixed-fixed condition (0.5) and fixed-pinned condition (0.7). This assumption was made to approximate the influence of the perforation radius *R* at the ends of the struts.

The out-of-plane compressive strength can be evaluated by the following equation:

(11) $$\sigma_{z}=\frac{F_{z}}{A_{s}}$$

where $F_{Z}=\frac{F_{cr}2\cos\left(A\right)}{\sin\left(B\right)}$ and $A_{s}=wt/4$.

Eventually, Equation (11) becomes:

(12) $$\sigma_{z}=\frac{2\frac{\pi^{2}EI_{2–3}\cos A}{{\left(0.6l_{0}\right)}^{2}}}{wt\sin B}$$

## 4. Experimental Approach

The experimental investigation aims to validate the analytical model defined for computing the strength and stiffness of the pyramidal cellular structure.

### 4.1. Manufacturing of Specimens

The specimens for the pyramidal structure were created by using a stainless-steel type 304 (E=187,000 MPa) sheet metal with a thickness of 0.25 mm.

The perforations were generated on a water jet cutting machine Maxiem 1530, Figure 5a, equipped with a 20 HP hydraulic pump, which can sustain a constant water pressure of 3500 bar.

**Figure 5:**
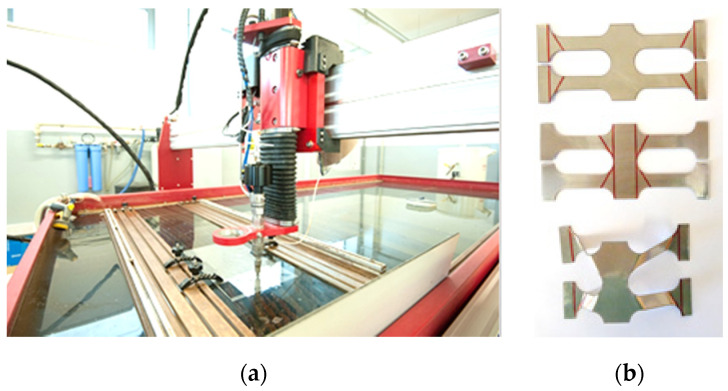
(**a**) Manufacturing method of the perforated profile (**b**) specimens used for the experimental testing.

The expansion angle *A* was marked and the bend lines required for initiating the expansion process were applied on the perforated profile, Figure 5b. The unit cells were afterwards expanded at the gauge dimensions, calculated using the equations presented in Table 1.

### 4.2. Investigated Geometric Configurations

The investigated geometric configurations of the pyramidal cellular structure were obtained by varying the parameter R=[3, 4, 5] mm resulting in the variation of the expansion angle A (Figure 2) while keeping constant the parameters B=60°, $l_{1}=10\;\mathrm{mm}$, $l_{0}=15\;\mathrm{mm}$ and c=15 mm, thus resulting in a number of three configurations named C1 ÷ C3, shown in Table 2.

After expanding the unit cells, they were individually glued to 1 mm thickness steel plates to fix the movement of the struts during compression, therefore limiting the influence of the edge effects. The adhesive used was a bi-component, epoxy based one, Araldite 2015, produced by Huntsman.

### 4.3. Experimental Protocol

The compression tests were performed on an Instron 3360 testing unit and were displacement driven with a constant crosshead speed of 3 mm/min. The load was measured using a 5 kN load cell. The compressive stress was calculated by dividing the measured load by the surface area of the unit cell $\left(w\times t\;{\mathrm{mm}}^{2}\right).$ The compressive strain was computed by dividing the crosshead displacement by the core initial height.

The out-of-plane elastic modulus was determined on the slope of the stress-strain curve as $E_{z}=\sigma_{z}/\varepsilon_{z}$.

## 5. Results

The out-of-plane compression tests have provided values for the strength ($\sigma_{z}$) and effective elastic modulus ($E_{z}$) for a single unit cell with the strut inclination angle equal to 60°. A comparison between experimental and analytical results is presented in Table 3.

As expected, the registered experimental values for the maximum strength and stiffness are slightly lower than the theoretical ones, since the analytical model does not take into consideration the geometric imperfections of the cell’s struts obtained during the manufacturing process.

In addition, the value for the bend radii, depicted in Figure 1 numbered 3, is equal to zero, while, in the case of the experimental testing, the bends performed have had a radius up to 1 mm. This radius contributes in avoiding the crack propagation in the sheet metal during the expansion process.

**Figure 6:**
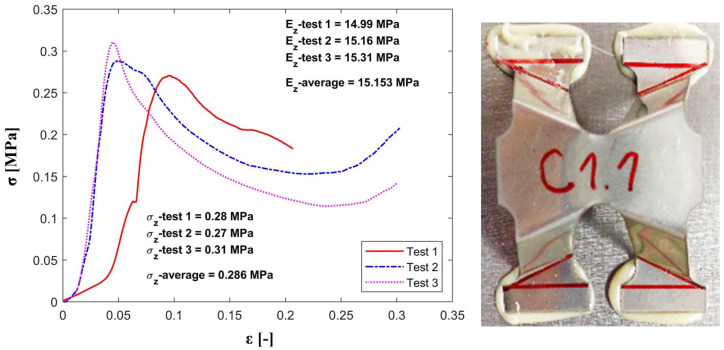
Stress-strain plot for the C1 configuration under out-of-plane compression.

**Figure 7:**
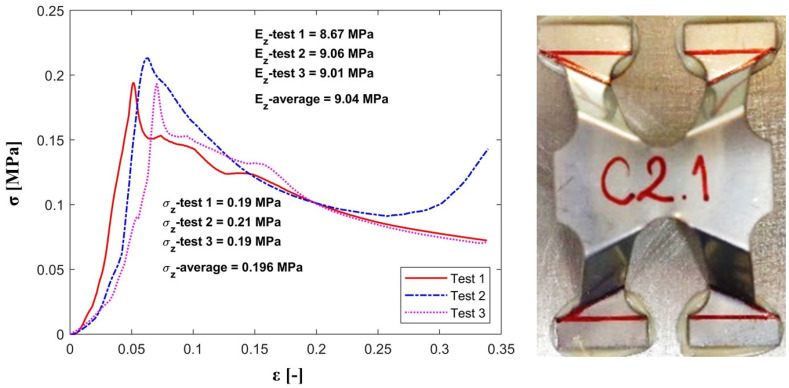
Stress-strain plot for the C2 configuration under out-of-plane compression.

**Figure 8:**
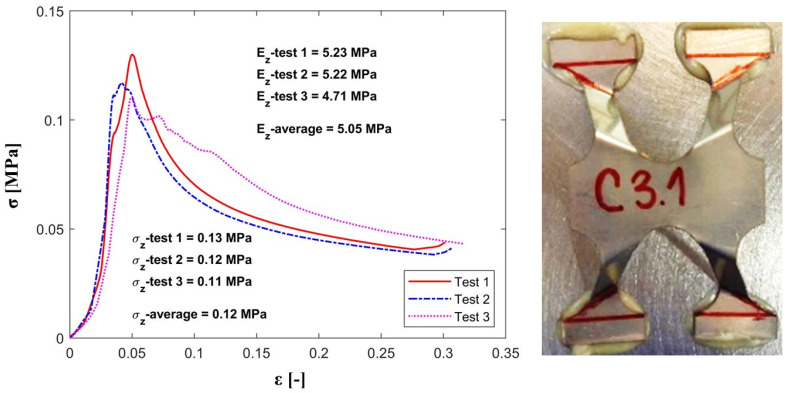
Stress-strain plot for the C3 configuration under out-of-plane compression.

Figure 6, Figure 7 and Figure 8 show the values for stiffness and strength for the samples as well as the mean value for all the configurations subjected to experimental testing (C1, C2 and C3).

The comparative study shows that both models, analytical and experimental, follow the same path which outlines that the analytical model was successfully validated through the experimental testing.

## 6. Parametric Study

Based on the validated analytical model, the out-of-plane mechanical properties can be studied while varying the geometric parameters.

**Figure 9:**
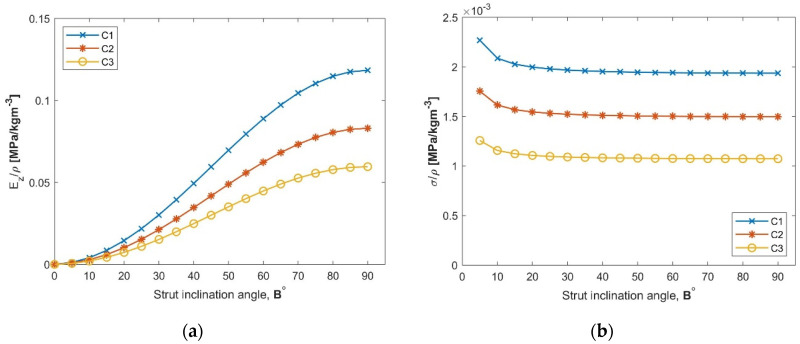
(**a**) Normalized compression stiffness and (**b**) normalized compression strength for the configurations subjected to study.

Figure 9 shows the evolution of the specific compressive stiffness and strength, $E_{z}/\rho$ and $\sigma_{z}/\rho$, for the investigated structure. The fixed parameters of the structure were $l_{0}=15\;\mathrm{mm}$, c=15 mm, $l_{1}=10\;\mathrm{mm}$ while the strut inclination angle varied in the range of [0°–90°]. The radius of the perforation was different for each of the tested configurations; R=3 mm for C1, R=4 mm for C2 and R=5 mm for C3. The expansion angle differed between the three configurations as well since its value is dependent on the perforation radius.

On the one hand, as expected, the stiffness of the pyramidal structure is significantly influenced by an increase of the strut inclination angle *B*, Figure 9a. On the other hand, it results that the specific stiffness will decrease with the increase of the perforation radius *R* (which turns into an increase of the angle *A*).

The strength of the structure, Figure 9b, decreases together with an increase of the perforation radius, R, and, therefore, with an increase of the angle *A*. The difference between the three configurations is caused by the slenderness of the cell’s strut. The highest values are recorded by the C1 configuration, where the radius of the perforation has the lowest value, R=3 mm.

## 7. Discussion

**Figure 10:**
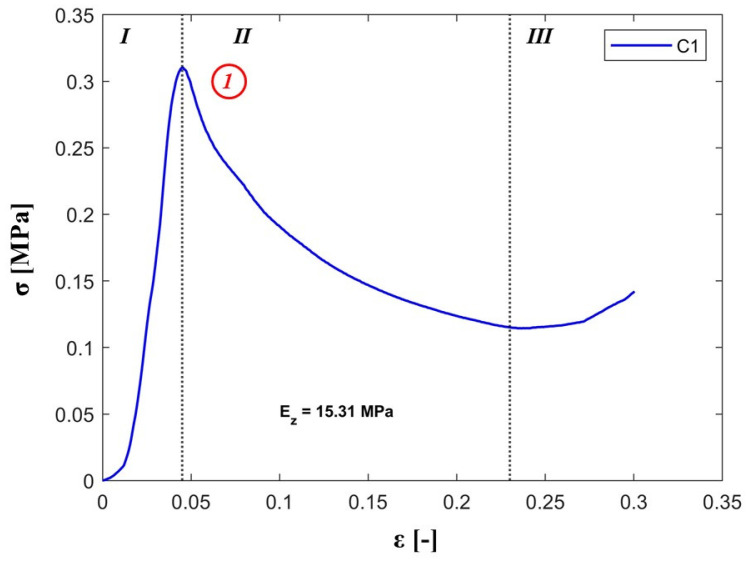
Stress-strain flow and characteristics of the investigated pyramidal cellular structure.

The typical out-of-plane nominal stress-strain response for the pyramidal structure is shown in Figure 10. It can be observed that the structure exhibits typical characteristics of corrugated cellular structures: a region of elastic response (I), peak strength (1) followed by a plastic region represented by a drop in flow typical to Euler’s buckling of the struts (II). The graph ends with a hardening region associated with core densification (III) [22].

Analyzing the results presented in Table 3, it can be concluded that $E_{z}$ decreases proportionally with the increase in value of the radius of the perforation, the major reason being the resulting slenderness of the cell’s strut.

**Figure 11:**
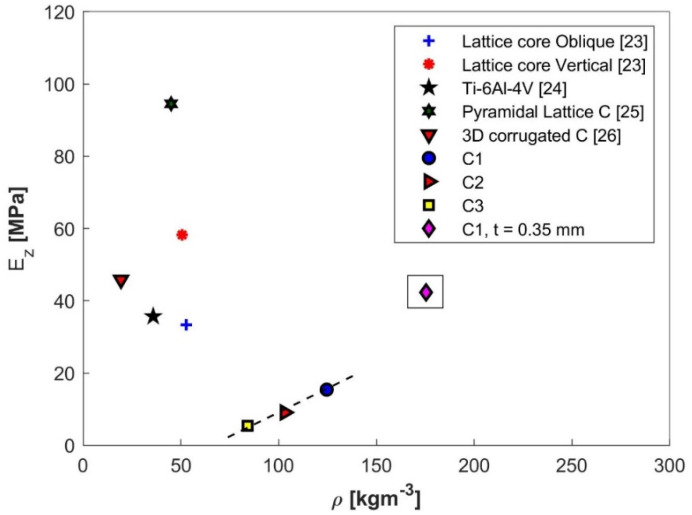
Out-of-plane stiffness as function of core density for several types of cellular structures.

Having the out-of-plane stiffness of the investigated structure defined and validated, its performance has been compared with a selection of different core types, as shown in Figure 11.

The pyramidal structure did not show better compressive stiffness performance as compared to the other structures taken into consideration. Despite this, it is relevant to mention that the thickness of the base material was 0.25 mm, significantly reduced from the other studied structures (e.g., 0.9 mm for the carbon fiber/epoxy lattice core oblique and lattice core vertical developed by J. Xiong et al. [23] and 0.635 for the titanium alloy structure presented by Queheillalt and Wadley [24]). The reduced thickness of the parent material made the pyramidal structure more susceptible to buckling. However, by taking into consideration a base material thickness to 0.35 mm and computing the new value based on Equation (9), the stiffness of the structure became 42.36 MPa for a density of 175 kg·m^−3^, marked in the rectangle on the graph. Through this configuration, the novel structure reached half the stiffness value compared to that of the pyramidal lattice C developed by Ming Li et al. [25] with a significantly higher density. The possibility of maintaining/increasing the stiffness while reducing the structure’s density could be obtained by further applying both perforating and embossing operations on the struts in order to increase their second moment of inertia or by replacing the stainless steel with a low density material (e.g., aluminum).

**Figure 12:**
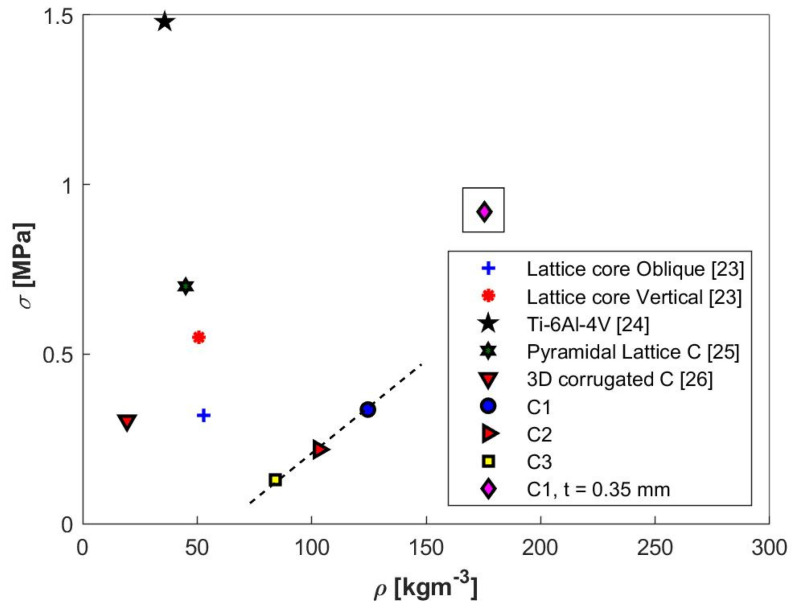
Strength as function of core density for several types of cellular structures.

The out-of-plane strength as a function of core density is illustrated in Figure 12. The highest performance with respect to strength has been registered for the C1 configuration with a maximum stress of 0.33 MPa. As a result, the newly developed structure is ranked above the lattice core oblique corrugation with a value of 0.32 and the 3D corrugated C presented by Jian Xiong et al. [26] but at a higher density.

If the material thickness used for the C1 configuration is increased from 0.25 mm to 0.35 mm, this brings the investigated structure to a compression strength of 0.92 MPa with a density of 175 kg·m^−3^. Perforating and embossing operations on the struts may be a solution for increasing compressive strength performance.

The value of the internal angle *A* has been considered to be a function of the radius *R* (Table 1). This assumption turned out to be a limitation with respect to the performance of the structure (both stiffness and strength) because to increase the angle *A*, one needs to increase the value of *R*, which results in a slender strut. Further investigations should consider the angle *A* independent from the radius *R*.

In the current study, the mechanical behavior of the structure was linked to the characteristics of the material the structure was made of. Therefore, for different values of *E* and *G* (longitudinal and transversal stiffness moduli), the structural behavior will change accordingly. Furthermore, the developed model has been proven to work for the investigated geometry. If a bigger or a smaller scale are considered, additional effects might have to be investigated. Temperature effects are also not taken into consideration within this study, but it is a topic of interest for further investigations. In particular, the behavior of the adhesive should be considered if the temperature varies significantly.

## 8. Conclusions

The main purpose of this research was to evaluate the mechanical properties of a novel pyramidal cellular core obtained through a mechanical expansion process for the construction of sandwich panels. A theoretical model has been developed to define the out-of-plane compressive behavior. Expressions for computing the maximum strength, and stiffness were defined with the help of the developed analytical model which was validated through experimental testing.

The following conclusions can be drawn regarding the proposed pyramidal structure:

- The present pyramidal cellular core has the potential to compete with other cellular core concepts
- A decreased internal angle A increases both the out-of-plane stiffness and out-of-plane strength, but increases the core density
- Increased overall structural performance (both stiffness and strength) may be achieved by considering the angle *A* independent from the radius *R*.
- The specific stiffness and strength behavior can be improved by using low-density materials or by increasing the second moment of inertia of the struts, for example through embossing operations.

## Figures and Tables

**Table 1:** Interdependence between geometric parameters for the investigated topology.

Geometric Parameter	Expression
Expansion angle	$A=arctan\left(\frac{2R}{c}\right)$
Length of the unit cell	$w=2l_{1}+2l_{o}cos\left(B\right)+ctan\left(A\right)+2R$
Width of the unit cell	$t=2c+z+l_{0}\sin\left(B\right)\tan\left(A\right)$
Height of the unit cell	$h=2g+l_{0}sin\left(B\right)$

**Table 2:** Configurations and dimensions of the samples for the compression tests.

Config.	*w* [mm]	*t* [mm]	*h* [mm]	*B* [°]	*A* [°]
C1	47	37	13.3	60	21.8
C2	51	38	13.3	60	28.1
C3	54	39	13.3	60	33.7

**Table 3:** Analytical and experimental results for the out-of-plane compression.

Config	Analytic		Experimental	
	$E_{z}\;\left[MPa\right]$	$\sigma_{z}\;\left[MPa\right]$	$E_{z}\;\left[MPa\right]$	$\sigma_{z}\left[MPa\right]$
C1	15.4	0.33	15.153	0.286
C2	9.08	0.22	9.04	0.196
C3	5.43	0.13	5.05	0.12
